# Natural Competence Is Common among Clinical Isolates of *Veillonella parvula* and Is Useful for Genetic Manipulation of This Key Member of the Oral Microbiome

**DOI:** 10.3389/fcimb.2017.00139

**Published:** 2017-04-20

**Authors:** Steven Knapp, Clint Brodal, John Peterson, Fengxia Qi, Jens Kreth, Justin Merritt

**Affiliations:** ^1^Department of Restorative Dentistry, Oregon Health and Science UniversityPortland, OR, USA; ^2^Department of Pediatric Dentistry, Oregon Health and Science UniversityPortland, OR, USA; ^3^Department of Microbiology and Immunology, University of Oklahoma Health Science CenterOklahoma, OK, USA; ^4^Department of Molecular Microbiology and Immunology, Oregon Health and Science UniversityPortland, OR, USA

**Keywords:** oral bacteria, natural competence, *Veillonella*, natural transformation, type II secretion, DNA uptake, type IV pili

## Abstract

The six *Veillonella* species found in the human oral cavity are among the most abundant members of the oral flora, occurring in both supra- and subgingival dental plaque as well as on the oral mucosa. Epidemiological data have also implicated these species in the development of the most common oral diseases. Despite their ubiquity, abundance, and ecological significance, surprisingly little is known about *Veillonella* biology, largely due to the difficulties associated with their genetic manipulation. In an effort to improve genetic analyses of *Veillonella* species, we isolated a collection of veillonellae from clinical plaque samples and screened for natural competence using a newly developed transformation protocol. Numerous strains of *V. parvula* were found to exhibit a natural competence ability that was highly influenced by growth medium composition. By exploiting this ability, we were able to utilize cloning-independent allelic exchange mutagenesis to identify the likely source of DNA uptake machinery within a locus homologous to type II secretion systems (T2SS). Interestingly, *V. parvula* natural competence was found to exhibit a clear hierarchy of preference for different sources of DNA (plasmid < PCR product < genomic DNA), which is unlike most naturally competent species. Genomic comparisons with other members of the Veillonellaceae family suggest that natural competence is likely to be widely distributed within this group. To the best of our knowledge, this study is the first demonstration of natural competence and targeted allelic exchange mutagenesis within the entire Veillonellaceae family and demonstrates a simple and rapid method to study *Veillonella* genetics.

## Introduction

Of the 13 species in the genus *Veillonella* (Aujoulat et al., 2014), six are typically found in the oral cavities of humans (*V. atypica, V. dispar, V. parvula, V. denticariosi, V. rogosae*, and *V. tobetsuensis*), where they comprise significant fractions of the oral flora found within supra- and subgingival dental plaque as well as on the oral mucosa (Aas et al., 2005; Beighton et al., 2008; Haffajee et al., 2009; Zaura et al., 2009; Abusleme et al., 2013). Veillonellae are also among the most prevalent species detected in saliva and dental plaque (Keijser et al., 2008; Valm et al., 2011). The prevalence and abundance of these organisms in the oral cavity is a reflection of their prominent role within the microbial ecology of oral biofilms. Veillonellae are considered bridge species due to their diverse array of intergeneric coaggregation/coadhesion interactions as well as their ability to stimulate the growth of numerous organisms through metabolic complementation (Hughes et al., 1988; Palmer et al., 2006; Periasamy and Kolenbrander, 2009a,b, 2010; Zhou et al., 2016). For example, veillonellae likely play a crucial role in the removal of toxic organic acid metabolic waste products from biofilm communities, due to their unusual preference for organic acid carbon sources (Delwiche et al., 1985). Likewise, many fastidious periodontal pathogens have an obligate growth requirement for heme, which can be provided by the veillonellae through *de novo* synthesis (Zhou et al., 2016). This and the various other unusual aspects of *Veillonella* metabolism are presumably of particular importance to support the persistence of periodontopathogens before the onset of oral inflammatory disease (i.e., during oral health) (Palmer et al., 2006; Periasamy and Kolenbrander, 2009a,b, 2010; Zhou et al., 2016).

While *Veillonella* species (especially *V. parvula*) have been associated with the development of the most common oral diseases, such as caries (Becker et al., 2002; Kanasi et al., 2010; Tanner et al., 2011), endodontic infections (Sundqvist, 1992; Khemaleelakul et al., 2002), and periodontitis (Kamma et al., 1995; Tanner et al., 1996; Heller et al., 2012; Mashima et al., 2015), little is known at the molecular level about why these associations exist. One of the principal hindrances to progress in this area has come from the historical difficulties associated with the genetic manipulation of veillonellae. *Veillonella* was generally considered a genetically intractable genus until recently, as no targeted mutations had ever been successfully introduced into any of the *Veillonella* species. However, this changed several years ago, when the *V. parvula* strain PK1910 was demonstrated to be transformable via electroporation of isogenic genomic DNA (Liu et al., 2011). While this strain of *V. parvula* proved to be resistant to electrotransformation with all other sources of DNA, this was not the case for the *V. atypica* clinical isolate OK5, which was later used to develop the first *Veillonella* genetic system (Liu et al., 2012). More recently, the first targeted marked and unmarked mutations of *Veillonella* were introduced into strain OK5 using insertion duplication mutagenesis with a suicide vector (Zhou et al., 2015a,b, 2016). These studies have provided some of the first insights into *Veillonella* genetics and further demonstrated the feasibility of *Veillonella* genetic research.

Encouraged by these studies, we were curious whether the *Veillonella* genetic system could be further improved using natural competence, rather than electrotransformation. Natural competence offers distinct advantages over electrotransformation for genetic studies, as the DNA uptake machinery protects transforming DNA from restriction enzyme cleavage after import (Chen and Dubnau, 2003, 2004; Johnston et al., 2013), while cells that have entered the natural competence state also concurrently activate their recombination systems, conveniently further increasing the likelihood of obtaining the desired recombinant (Kidane et al., 2012; Straume et al., 2015). These features have made it possible to develop a variety of rapid and highly efficient cloning-independent mutagenesis and plasmid assembly techniques for some naturally competent species, such as *Streptococcus mutans* (Xie et al., 2011, 2013). Unfortunately, only a minority of species is known to be naturally competent. The principal difficulty in observing natural competence in new species is probably related to the highly disparate signals required to trigger competence gene expression. In most species, natural competence is transient and stringently regulated by secreted signal molecules and/or very specific environmental cues, such as chitin polymers in the case of *Vibrio* natural competence (Seitz and Blokesch, 2013; Fontaine et al., 2015; Matthey and Blokesch, 2016). While the signals triggering competence development can be highly species- and/or strain-specific, the competence machinery itself is quite similar among naturally competent bacteria, with most identified DNA uptake loci containing genes homologous to type II secretion systems (T2SS) as well as homologs of genes required for type IV pilin biogenesis (Averhoff and Friedrich, 2003; Chen and Dubnau, 2003, 2004). In the current study, we provide evidence that most veillonellae encode such competence loci and demonstrate how these systems may be exploitable for their facile genetic manipulation.

## Materials and methods

### Bacterial strains and growth conditions

*Escherichia coli* cultures were maintained at 37°C in Luria Bertani medium (LB; Difco, Sparks, MD) with aeration or grown aerobically on LB agar plates. All *Veillonella* cultures and agar plates were grown at 37°C in anaerobic conditions (85% nitrogen, 10% carbon dioxide, and 5% hydrogen) using either Todd Hewitt medium (Difco, Sparks, MD) supplemented with 0.3% wt/vol yeast extract (Fisher, Fair Lawn, NJ) and 0.6% sodium lactate (Spectrum Chemical, Gardena, CA) (together referred to as THL medium) or a novel medium referred to as SK medium. SK medium composition is as follows: 10 g L^−1^ tryptone (Fisher, Fair Lawn, NJ), 10 g l^−1^ yeast extract (Fisher, Fair Lawn, NJ), 0.4 g l^−1^ disodium phosphate (Fisher, Fair Lawn, NJ), 2 g l^−1^ sodium chloride (Fisher, Fair Lawn, NJ), and 10 ml l^−1^ 60% (wt/vol) sodium lactate (Spectrum Chemical, Gardena, CA). For antibiotic selection, cultures and/or agar plates were supplemented with the following antibiotics: 150 μg ml^−1^ ampicillin (*E. coli*), 9 μg ml^−1^ tetracycline (*Veillonella*), and 350 μg ml^−1^ spectinomycin (*Veillonella*).

### Isolation of *Veillonella* from clinical plaque samples

Prior to the study, the clinical sample collection and strain isolation protocol were reviewed by the Oregon Health and Science University Institutional Review Board (IRB) and deemed not human subjects research. All clinical specimens were collected by clinicians in the OHSU Pediatric Dental clinic using material generated during routine treatment procedures. Specimens were removed of all Protected Health Information (PHI) identifiers as part of the collection protocol. Clinical specimens were derived from a pediatric cohort and consisted of 67 unique buccal molar plaque samples. Initially, *Veillonella* clinical isolates were obtained using a previously published protocol (Liu et al., 2012). Briefly, plaque samples obtained from the clinic were vortexed to disperse the cells and then 100 μl was plated onto TH plates containing a confluent lawn of *S. mutans* strain UA159 previously grown for 24 h. Plates were incubated anaerobically at 37°C for 48 h. before inspecting the plates for the presence of colonies forming on top of the *S. mutans* lawn. Cells from candidate colonies were observed by microscopy for the expected cellular morphology. Promising clones were restreaked and then further tested by 16S rRNA sequencing and, in some cases, *rpoB* PCR amplification. In an effort to improve the efficiency of *Veillonella* isolation, a new enrichment strategy was developed and implemented. For this approach, each unique clinical plaque sample was used to inoculate two separate 1 ml cultures of SK medium +15.6 μg ml^−1^ erythromycin as well as SK medium +0.5 μg ml^−1^ chloramphenicol. Antibiotic enrichment cultures were incubated until reaching stationary phase (typically 24 h) and then quadrant streaked onto SK agar plates. Cells from isolated colonies were observed by microscopy for the expected cellular morphology. Candidate clones were restreaked and then further tested by 16S rRNA sequencing and, in some cases, *rpoB* PCR amplification.

### *Veillonella* species identification

All primers used in the study are listed in Table 1. Pure culture isolates of candidate *Veillonella* species were tested by amplifying the 16S rRNA gene using PCR and the primers 16S Uni F/R. The resulting PCR products were purified using the DNA Clean and Concentrator kit (Zymo, Irvine, CA) and then sequenced using the 16S Uni F/R primers. Species assignments were made by performing a BLASTN search of 16S sequence data in both the Human Oral Microbiome Database (www.homd.org) and the Green Genes database (greengenes.lbl.gov). In some cases, it was necessary to further verify species assignments using PCR amplification of the *Veillonella rpoB* gene (Igarashi et al., 2009). 5 unique *rpoB* forward primers (DENF, PARF, ROGF, ATYF, and DISF) were combined in a single PCR reaction with the *Veillonella rpoB* universal reverse primer VR. Based upon amplicon sizes, it was possible to determine the species of the tested isolates. All identified *Veillonella* clinical isolates are listed in Table 2.

**Table 1:** **Primers used in this study**.

**Primer**	**Sequence 5′ → 3′**	**Purpose**
16S Uni F	AGAGTTTGATCCTGGCTCAG	Species identification
16S Uni R	ACGGMTACCTTGTTACGACTT	Species identification
DENF	GAAAGAAGCGCGCACCGACAG	Species identification
PARF	GAAGCATTGGAAGCGAAAGTTTCG	Species identification
ROGF	ATTGCAGAAGATGTAACAGTAAGC	Species identification
ATYF	TCTCTTGTTGAAGAATTAGAACGC	Species identification
DISF	AACGCGTTGAAATTCGTCATGAAC	Species identification
VR	GTGTAACAAGGGAGTACGGACC	Species identification
pBS F	CAGCAATAAACCAGCCAGCC	pBSJL1 detection
pBS R	CGCCGCATACACTATTCTCAG	pBSJL1 detection
TetM F	AGTAAAATGCAGGCGAGTGAAG	*recA* & CGL mutagenesis
TetM R	CCCAGGACACAATATCCACTTG	*recA* & CGL mutagenesis
gspE up F3	GTCGTCTTATGGGGAATGAAGG	CGL mutagenesis
Tmp up R3	**CTTCACTCGCCTGCATTTTACT** ACACCACAACAGGAACAAGC	CGL mutagenesis
4715 dn F	**CAAGTGGATATTGTGTCCTGGG** GCAAGGGGGCTTAAATGGATTG	CGL mutagenesis
4725 dn R	TCTATTTCTATACGGCGTGAAGG	CGL mutagenesis
gspE up F4	GGCTGTCAGATGGCAGTTATTC	CGL mutagenesis
4725 dn R2	GGACCACCACCACGTTG	CGL mutagenesis
gspF dn F3	GGACCACCACCACGTTG	CGL mutagenesis
pulO dn R3	TCCCCTCCAAATCAACTGATAGAG	CGL mutagenesis
gspE up F3	GTCGTCTTATGGGGAATGAAGG	CGL mutagenesis
gspF dn F2	AAGTACATCGTTGGCTTTGGC	CGL mutagenesis
pulO dn R2	**CTTCACTCGCCTGCATTTTACT** GTCCCTTTCATACCTTGTCCTT	CGL mutagenesis
pulO dn F2	**CAAGTGGATATTGTGTCCTGGG** GTGTTGGTTCTCTCTATCTCTTACTAAC	CGL mutagenesis
964 dn R	CCTTCATATCAAAATCGTGAGGATCA	CGL mutagenesis
recA up F	AGTACTGGTGGGTTAGGACC	*recA* mutagenesis
recA up R	**CTTCACTCGCCTGCATTTTACT** CAACGCTGCTTGTCTACCATC	*recA* mutagenesis
recA dn F	**CAAGTGGATATTGTGTCCTGGG** CCGCTGAATTTGATTTGATGTATGG	*recA* mutagenesis
recA dn R	CAGCCTCTTGCTCAGCTTG	*recA* mutagenesis
recA up F2	CGCTCAAGATACAACAAGTTATGGC	*recA* mutagenesis
recA dn R2	ACGAGCCACATCAACACCG	*recA* mutagenesis

*Sequences in bold are complementary to the tetM tetracycline resistance cassette*.

**Table 2:** **Species identification of *Veillonella* clinical isolates**.

**Strain**	**16S**	***rpoB***
SKV1	*V. parvula*	*V. parvula*
SKV3	*V. atypica*	
SKV4	*V. atypica*	
SKV8	*V. atypica*	
SKV9	*V. parvula/dispar*	*V. parvula*
SKV10	*V. parvula*	*V. parvula*
SKV11	*V. dispar/atypica*	*V. atypica*
SKV12A	*V. parvula*	*V. parvula*
SKV14	*V. atypica*	
SKV15	*V. atypica*	
SKV16	*V. atypica*	
SKV17A	*V. atypica*	
SKV17B	*V. parvula/dispar*	*V. parvula*
SKV21	*V. parvula/dispar*	*V. parvula*
SKV23	*V. atypica*	
SKV24B	*V. parvula*	*V. parvula*
SKV25	*V. atypica*	
SKV26	*V. dispar*	*V. dispar*
SKV29	*V. atypica*	
SKV30	*V. atypica*	
SKV31B	*V. parvula/dispar*	*V. parvula*
SKV32	*V. atypica*	
SKV36B	*V. atypica*	
SKV38	*V. parvula/dispar*	*V. parvula*
SKV40B	*V. atypica*	
SKV43	*V. parvula*	*V. parvula*
SKV44	*V. parvula*	*V. parvula*
SKV45A	*V. atypica*	
SKV49B	*V. denticariosi*	
SKV52	*V. parvula*	*V. parvula*
SKV53	*V. atypica*	
SKV54	*V. atypica*	
SKV55	*V. atypica*	
SKV60	*V. atypica*	
SKV61	*V. atypica*	
SKV62	*V. atypica*	
SKV63	*V. atypica*	
SKV64A	*V. atypica*	
SKV65A	*V. dispar*	*V. dispar*
SKV65B	*V. atypica*	
SKV65C	*V. parvula/dispar*	*V. parvula*
SKV66	*V. parvula/dispar*	*V. parvula*

### Shuttle plasmid pBSJL1 transformation

*V. parvula* strains SKV17B and SKV38 were streaked onto both SK and THL plates and incubated anaerobically at 37°C for 48 h. Cells were scraped from each of the plates and resuspended in 1 ml of their respective liquid media (SK or THL) and then vortexed to disperse. The cell densities of the cultures were subsequently adjusted to OD_600_ 0.4. Next, 10 μl of the cell suspensions was spotted onto the respective agar plates (SK or THL) and then immediately followed by adding an additional 800 ng of the *E. coli-Veillonella* shuttle plasmid pBSJL1 (dissolved in 3 μl TE buffer) (Liu et al., 2012) to the spots. Plates were allowed to dry in the anaerobic chamber before incubating anaerobically at 37°C for 48 h. Afterward, cells growing in the 10 μl spots were scraped from the plates and resuspended in 250 μl of their respective liquid media (SK or THL) and then vortexed to disperse. 10 μl of each cell suspension was spotted onto SK plates supplemented with tetracycline and incubated anaerobically at 37°C for an additional 48 h. Candidate pBSJL1 transformants of SKV17B and SKV38 were inoculated into SK medium supplemented with tetracycline and then tested for the presence of pBSJL1 using PCR and the primers pBS F/R.

### Extraction of pBSJL1 from *V. parvula*

Plasmid extraction from *V. parvula* was performed using the typical alkaline lysis solutions I, II, and III (Green and Sambrook, 2016) with minor modifications as described below. 12 ml cultures of SK medium + tetracycline were inoculated with pBSJL1 transformants of *V. parvula* strains SKV17B and SKV38 and grown to stationary phase. Separate 12 ml non-selective SK medium cultures were also inoculated with wild-type plasmid-free SKV17B and SKV38 to serve as negative controls. The cultures were each divided into 3 × 4 ml aliquots and pelleted by centrifugation. Cells were resuspended in 250 μl of solution I containing 2.5 mg lysozyme and incubated in a 37°C water bath for 1.5 h. with shaking every 30 min. Next, 250 μl of solution II was added to lyse the cells followed by an additional 350 μl of solution III to neutralize the lysate. Cellular debris was pelleted by centrifugation at 16,000 × g for 8.5 min. and the cleared supernatants were collected. Supernatants were treated with phenol/chloroform solution, precipitated with 2-propanol, and resuspended in 70 μl of TE buffer. Purified pBSJL1 was used to retransform *E. coli* along with the negative control samples prepared from wild-type plasmid-free SKV17B and SKV38.

### Construction of *V. parvula recA* and competence locus deletion mutants

All primers used for mutation construct assembly are listed in Table 1. All primers and mutagenesis constructs were designed using Serial Cloner software (https://serial-cloner.en.softonic.com). Allelic exchange mutation constructs were assembled using overlap extension PCR ligation (Xie et al., 2013) with Phusion DNA polymerase (New England Biolabs, New Ipswich, MA) and strain SKV38 chromosomal DNA and pBSJL1 as PCR templates. The *recA* construct was assembled by first amplifying the upstream and downstream homologous fragments from strain SKV38 using the primers recA up F/R and recA dn F/R. 0.5 μl of both resulting PCR amplicons was mixed with a PCR amplicon of the *tetM* tetracycline resistance cassette amplified using pBSJL1 as a template and the primers TetM F/R. Regions of complementarity between the *recA* homologous fragments and the *tetM* amplicon facilitated their subsequent PCR ligation using the primers recA up F/recA dn R. A similar strategy was used to assemble constructs creating both large and small deletions within the competence gene locus. For the large deletion, about 5.7 kb of the 8 kb locus was deleted. Primers used to amplify the upstream and downstream homologous fragments for this construct were gspE up F3/TMP up R3 and 4715 dn F/4725 dn R, respectively. For the small deletion, 1 kb was deleted from the middle of the locus. The upstream and downstream homologous fragments were amplified with the primers gspF dn F2/pulO dn R2 and pulO dn F2/964 dn R, respectively. 0.5 μl of the resulting PCR amplicons was mixed with a PCR amplicon of the *tetM* tetracycline resistance cassette amplified with the primers TetM F/R. Regions of complementarity between the competence locus homologous fragments and the *tetM* amplicon facilitated their subsequent PCR ligation using the primers gspE up F3/4725 dn R for the large deletion construct and gspF dn F2/964 dn R for the small deletion construct. The resulting PCR products were transformed into wild-type *V. parvula* strain SKV38 and selected on agar plates supplemented with tetracycline. Additional competence locus mutation constructs creating large deletions were assembled using template gDNA extracted from a confirmed competence gene locus large deletion mutant and amplifying with the primers gspE up F4/4725 dn R2 as well as gspE up F3/4725 dn R. These PCR products were subsequently transformed into wild-type *V. parvula* strain SKV38 and selected on agar plates supplemented with tetracycline. Additional competence locus mutation constructs creating small deletions were assembled using template gDNA extracted from a confirmed competence gene locus small deletion mutant and amplifying with the primers gspF dn F3/pulO dn R3 as well as gspE up F3/4725 dn R. These PCR products were subsequently transformed into wild-type *V. parvula* strain SKV38 and selected on agar plates supplemented with tetracycline.

### Transformation of allelic exchange mutagenesis constructs and spectinomycin resistant mutant genomic DNA

The same natural transformation protocol described for pBSJL1 transformation was used to transform the allelic exchange mutagenesis constructs created by overlap extension PCR ligation. Transformation reactions both contained an equivalent molar ratio of a saturating amount of transforming DNA. For the *recA* construct 75 ng was transformed (7.5 μg ml^−1^ final concentration), while 145 ng of the competence locus construct was transformed (14.5 μg ml^−1^ final concentration). Transformants were selected on SK agar plates supplemented with tetracycline, confirmed via PCR, and finally tested for their natural competence abilities using the previously described natural transformation protocol. Wild-type and mutant strains were transformed with isogenic genomic DNA derived from a spontaneous spectinomycin resistant isolate of SKV38. The spectinomycin resistant mutant was isolated by plating 500 μl of a stationary phase culture of SKV38 onto SK agar plates supplemented with spectinomycin. 500 ng of spectinomycin resistant mutant genomic DNA (50 μg ml^−1^) was added to each transformation reaction and transformants were selected on SK agar plates supplemented with 350 μg ml^−1^ spectinomycin.

### Comparison of transformation efficiencies using plasmid, PCR product, and genomic DNA

The same previously described natural transformation protocol was used to transform wild-type SKV38 with either 300 ng pBSJL1, 145 ng of competence locus deletion PCR product, or 500 ng genomic DNA. To assess transformation efficiency, cells were plated on both non-selective and selective SK agar plates. Transformation efficiency is defined as the ratio of transformants to total CFU. Data are expressed as the average and standard deviations of at least three independent experiments performed in triplicate.

### Transformation of *V. parvula* strains with genomic DNA

The same natural transformation protocol described for pBSJL1 transformation was used to transform a panel of wild-type *V. parvula* strains with 500 ng genomic DNA derived from a tetracycline resistant competence locus deletion mutant of strain SKV38. In some cases, it was necessary to transform gDNA derived from a spontaneous spectinomycin resistant mutant of strain SKV38 due to the inherent tetracycline resistance of the parent strain. In such cases, 500 ng of gDNA conferring spectinomycin resistance was transformed and the transformants were selected on agar plates supplemented with spectinomycin. Data are presented as the number of tetracycline resistant transformants generated from 10 μl of transformation reaction cell suspension. Data are expressed as the average and standard deviations of three independent experiments performed in triplicate.

## Results

### Identification of naturally competent *V. parvula* strains from clinical plaque samples

**Figure 1:**
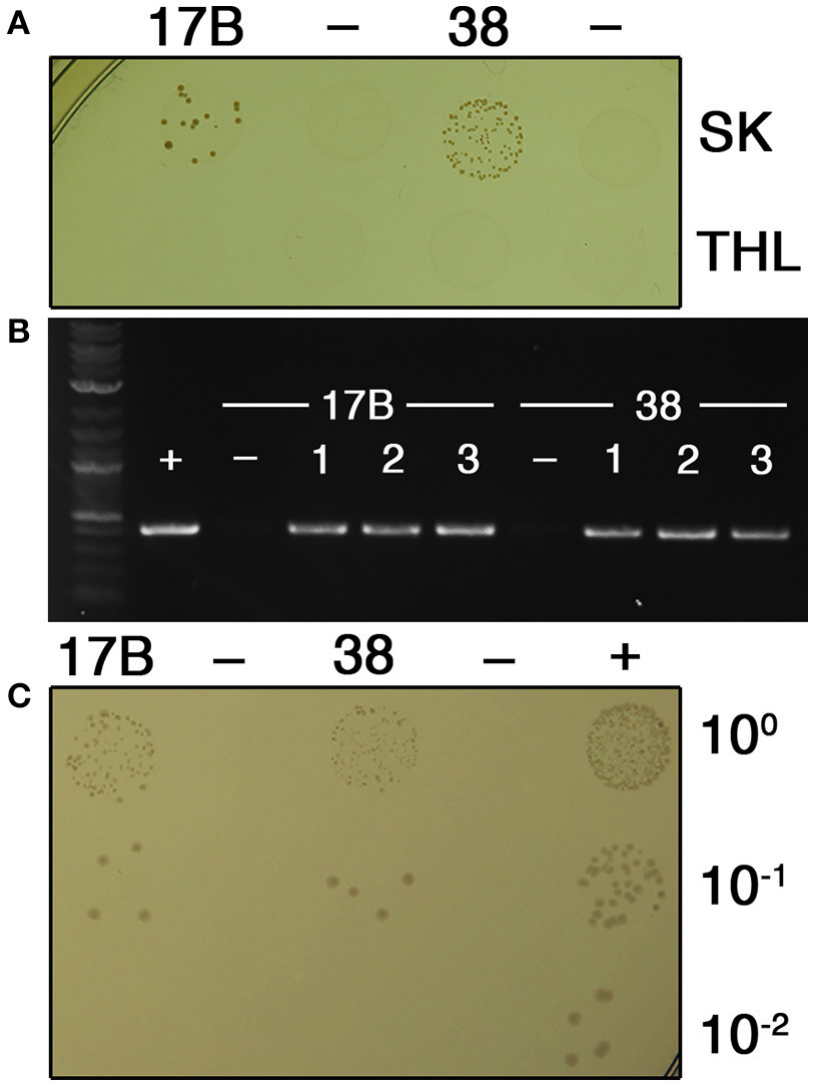
***V. parvula* clinical isolates exhibit natural competence**. **(A)** *V. parvula* strains SKV17B and SKV38 were transformed with the *E. coli-Veillonella* shuttle vector pBSJL1 in either SK medium (top row) or THL medium (bottom row). Ten microliters of the transformation reaction was spotted onto selective medium to isolate transformants. Samples from left to right are: SKV17B + pBSJL1, SKV17B (no DNA), SKV38 + pBSJL1, and SKV38 (no DNA). **(B)** Three CFU from the SKV17B and SKV38 transformation plates were tested for the presence of pBSJL1 using plasmid-specific primers. The untransformed parental strains were included as negative controls. **(C)** Plasmid extraction was performed on confirmed *V. parvula* transformants as well as the untransformed parental strains and then the resulting samples were transformed into *E. coli*. Plasmid samples from left to right were purified from the following sources: SKV17B + pBSJL1, SKV17B, SKV38 + pBSJL1, SKV38, and pBSJL1 purified from *E. coli*.

Recently, several mutations have been introduced into *V. atypica* strain OK5 using electrotransformation of the suicide vector pBST (Zhou et al., 2015a,b, 2016). These studies successfully demonstrated the potential genetic tractability of veillonellae. While this approach has proven to be a reliable mechanism to generate targeted mutations in strain OK5, the entire procedure can be laborious, and thus far, only single crossover insertion duplication mutations have been achieved despite previous attempts to create double crossover allelic exchange mutations (Zhou et al., 2015a). One of the principal benefits of allelic exchange mutagenesis is that the constructs can be easily assembled using solely PCR, circumventing the cloning requirement for suicide vector construction. This greatly increases the speed at which constructs can be assembled and avoids issues related to construct toxicity in *E. coli*. Since it was already established that allelic exchange mutagenesis is ineffective via electrotransformation of OK5, we were curious whether veillonellae might be naturally transformable, and thus, likely amenable to allelic exchange mutagenesis. To test this, we first isolated a collection of veillonellae from clinical plaque samples in a pediatric cohort from the OHSU pediatric dental clinic. In total, 67 unique buccal molar plaque specimens were collected from separate patients, resulting in a total of 43 unique *Veillonella* isolates. Initially, we employed a previously published *Veillonella* isolation technique in which strains are isolated on confluent lawns of *S. mutans* (Liu et al., 2012). While this approach did yield isolates, our success rate was lower than desired. Therefore, we developed a new simple and efficient protocol to improve the reliability of *Veillonella* isolation from plaque samples. As described in Materials and Methods, clinical specimens are first directly enriched for veillonellae by incubating in duplicate cultures of SK medium + chloramphenicol and SK medium + erythromycin. We found this single enrichment step to substantially increase the proportion of *Veillonella* in one or both cultures. Consequently, we were able to simply streak the enrichment cultures on non-selective plates and identify candidate veillonellae from the resulting colonies. Of the 43 unique isolates, we obtained multiple strains of the three most common *Veillonella* species found in dental plaque (*V. atypica, dispar*, and *parvula*) as well as a lone isolate of the less common species *V. denticariosi* (Table 2). Species assignments were based upon 16S rRNA sequence data. Since it was difficult to definitively distinguish between *V. dispar* and *V. parvula* using 16S rRNA sequences, each of these isolates was further confirmed by amplification of the *rpoB* gene (Igarashi et al., 2009). Next, each of the strains was tested for natural competence by transforming the *E. coli-Veillonella* shuttle vector pBSJL1 using an agar plate transformation approach with SK and THL media. Surprisingly, two strains of *V. parvula* (SKV17B and SKV38) yielded candidate transformants using SK medium, whereas no candidates were detected from any of the strains using THL medium (Figure 1A). Three candidates from the SKV17B and SKV38 transformations were further tested for the presence of pBSJL1 using plasmid-specific PCR primers and all appeared to contain the vector (Figure 1B). As a final confirmation, plasmid DNA was extracted from these transformants and then retransformed into *E. coli*, resulting in the expected acquisition of antibiotic resistance (Figure 1C).

### Allelic replacement mutagenesis using natural competence

**Figure 2:**
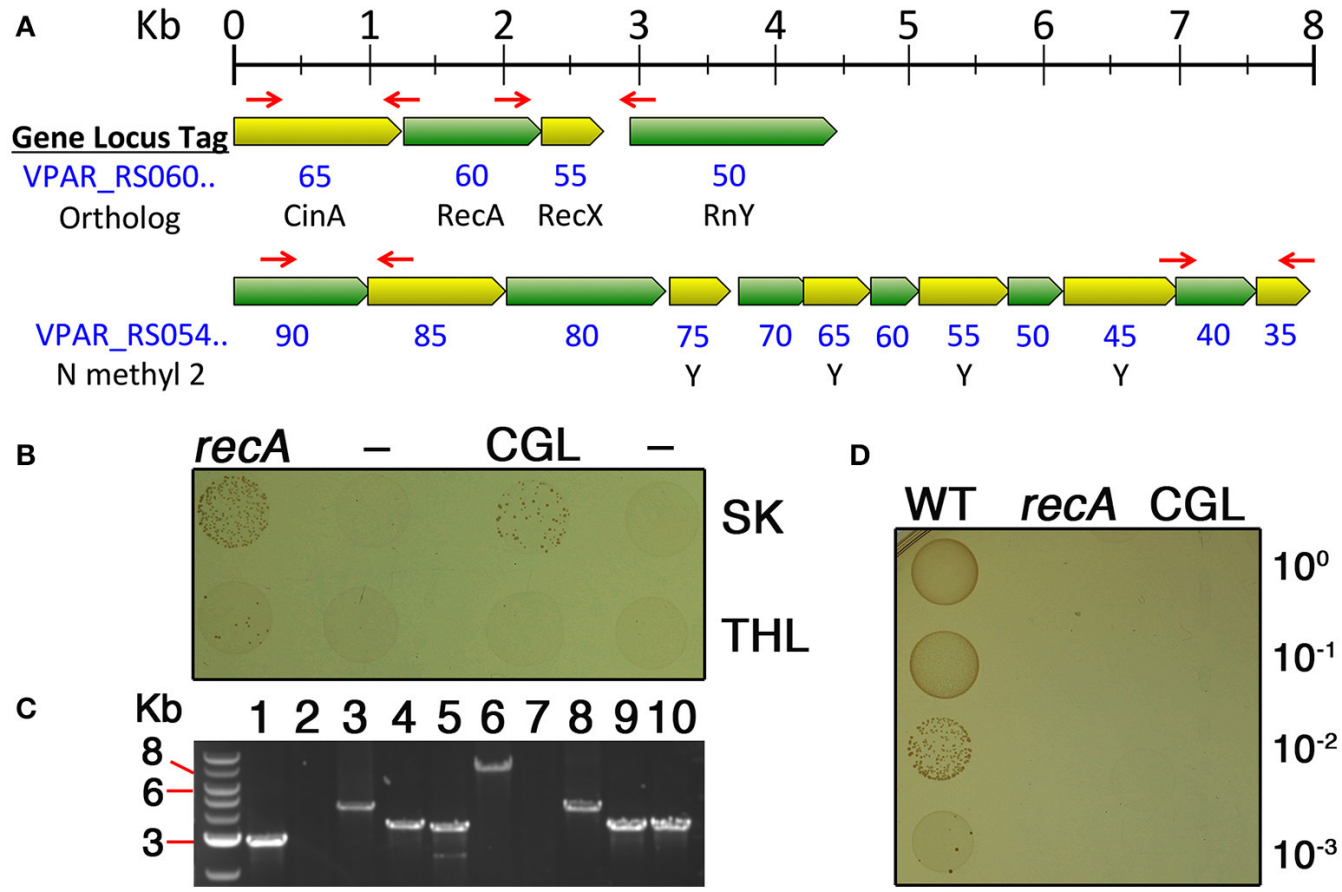
**Allelic replacement mutagenesis of *recA* and a putative competence gene locus. (A)** Chromosome maps of regions targeted for allelic exchange mutagenesis. Open reading frames (ORFs) are all drawn to scale. Red arrows indicate the locations of primers used to amplify homologous fragments used for mutagenesis. For both loci, the region between the two primer sets was replaced with a *tetM* cassette. NCBI gene locus tags are written in blue with the final two digits of the locus tag listed directly below their respective ORFs. For the competence gene locus map, the bottom row (N methyl 2) indicates whether a putative A24 prepilin cleavage/N-terminal methylation motif is present within the ORF. This motif is commonly found among type IV-like prepilins. **(B)** *V. parvula* strain SKV38 was transformed using either SK medium (top row) or THL medium (bottom row) together with PCR products containing allelic exchange mutagenesis constructs for both *recA* and the competence gene locus (CGL). Ten microliters of the transformation reaction was spotted onto selective medium to isolate transformants. Samples from left to right are: SKV38 + Δ*recA* PCR, SKV38 (no DNA), SKV38 + ΔCGL PCR, and SKV38 (no DNA). **(C)** Transformants from both the Δ*recA* and ΔCGL transformations were tested via PCR to confirm the expected genotypes. Samples from left to right are: 1. wild-type gDNA + *recA* locus external primers (predicted size 2,846 bp); 2. wild-type gDNA + *tetM* primers (no predicted amplicon); 3. Δ*recA* gDNA + *recA* locus external primers (predicted size 4,176 bp); 4. Δ*recA* gDNA + *recA* locus upstream external primer/*tetM* reverse primer (predicted size 3,162 bp); 5. Δ*recA* gDNA + *tetM* forward primer/*recA* locus external downstream primer (predicted size 3,086 bp); 6. wild-type gDNA + CGL external primers (predicted size 7,602 bp); 7. wild-type gDNA + *tetM* primers (no predicted amplicon); 8. ΔCGL gDNA + CGL external primers (predicted size 3,938 bp); 9. ΔCGL gDNA + CGL upstream external primer/*tetM* reverse primer (predicted size 2,992 bp); 10. ΔCGL gDNA + *tetM* forward primer/CGL external downstream primer (predicted size 3,018 bp). **(D)** Confirmed deletion mutants of *recA* and the competence gene locus were transformed with gDNA derived from a spontaneous spectinomycin resistant mutant of strain SKV38. Ten microliters of the transformation reaction and three consecutive serial dilutions were spotted onto selective medium to isolate transformants. Samples from left to right are: wild-type (WT), Δ*recA* (*recA*), and ΔCGL (CGL).

Given the success with pBSJL1 transformation, we were next curious to test whether the same procedure could be used for targeted mutagenesis of *V. parvula* via allelic exchange mutagenesis with PCR products. We were particularly interested to mutagenize genes potentially responsible for exogenous DNA uptake. In most naturally competent bacteria, the DNA uptake machinery is encoded by loci containing genes homologous to those of T2SS and genes involved in type IV pilin biogenesis (Averhoff and Friedrich, 2003; Chen and Dubnau, 2003, 2004). A candidate locus was identified in the sequence data for the *V. parvula* genome reference strain DSM2008 (VPAR_RS05490—5435) (Figure 2A). The 12 genes in the locus appeared to be organized into one or two polycistronic operons. The first two genes in the locus (VPAR_RS05490 and 5485) encode putative traffic NTPases resembling those of T2SS with the second gene product exhibiting homology to PilT proteins. Most *bona fide* T2SS do not encode a second traffic NTPase, whereas this is a common feature of type IV pilin biogenesis loci in both Gram positive and Gram negative bacteria (Chen and Dubnau, 2004; Giltner et al., 2012). In addition, a variety of the downstream open reading frames (ORFs) between VPAR_RS05465—5435 have features that are highly reminiscent of type IV prepilins (Figure 2A). The ORFs in this locus were further analyzed using the PilFind webserver (http://signalfind.org/pilfind.html) (Imam et al., 2011) and a canonical type IV prepilin leader sequence was detected in VPAR_RS05465. This ORF is also located directly adjacent to the putative prepilin peptidase (VPAR_RS05470) likely responsible for cleaving such leader peptides (Giltner et al., 2012) (Figure 2A). These features were all suggestive of a type IV pilin biogenesis locus, many of which serve as competence loci (Chen and Dubnau, 2004; Giltner et al., 2012), and thus it was targeted for mutagenesis. As a control, we also targeted *recA* for mutagenesis, since this mutant was expected to lose its natural transformability due to severe impairments in homologous recombination. Allelic exchange mutagenesis constructs were assembled via PCR and transformed into SKV38 using both SK and THL media. Once again, SK medium yielded substantially higher numbers of transformants compared to THL (Figure 2B), further confirming our previous observations. Antibiotic resistant transformants were subsequently tested via PCR and confirmed to exhibit the expected mutant genotypes for both the *recA* and competence gene locus deletions (Figure 2C). Next, we assayed the natural competence abilities of the two allelic exchange mutant strains. Since only one antibiotic resistance cassette is available for veillonellae, we transformed genomic DNA (gDNA) derived from a spontaneous spectinomycin resistant mutant of SVK38. These transformants could then be selected on plates supplemented with spectinomycin. To our surprise, the wild-type control yielded a large number of transformants far beyond what was anticipated based upon the previous transformations with either pBSJL1 or PCR products (Figure 2D). However, this was not the case for both the *recA* and competence gene locus deletion strains, as no transformants were detected from either (Figure 2D). Thus, both deletions resulted in a minimum of 3-log reductions in transformability.

### *Veillonella* natural transformation exhibits an uptake bias for the source of DNA

**Figure 3:**
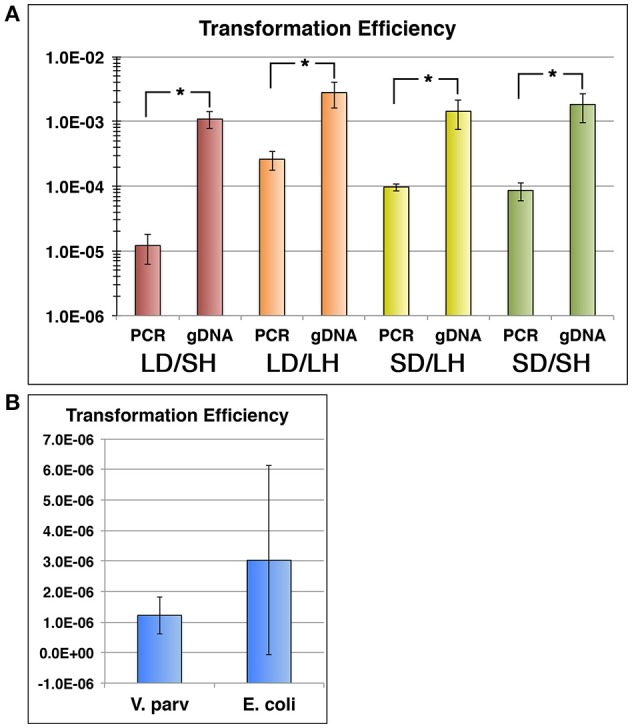
***Veillonella* natural transformation exhibits a distinct uptake bias. (A)** *V. parvula* SKV38 was transformed with both PCR product allelic exchange mutagenesis constructs targeting the competence gene locus as well as isogenic gDNAs derived from their respective mutant strains. Constructs contained various combinations of either large chromosomal deletions (LD) or small chromosomal deletions (SD) as well as large homologous fragments (LH) or small homologous fragments (SH). Construct designs are as follows. Red: large chromosomal deletion (~5.7 kb) and small homologous fragments (~0.9 kb), Orange: large chromosomal deletion (~5.7 kb) and large homologous fragments (>2.5 kb), Yellow: small chromosomal deletion (~0.4 kb) and large homologous fragments (>2.5 kb), and Green: small chromosomal deletion (~0.4 kb) and small homologous fragments (~0.9 kb). **(B)** *V. parvula* SKV38 was transformed with the *E. coli—Veillonella* shuttle vector pBSJL1 purified from either *V. parvula* or *E. coli*. All transformation efficiencies were determined by calculating the ratios of transformants to total CFU. The data are presented as the means ± standard deviations from at least three independent experiments performed in triplicate. ^*^P < 0.01 as determined by unpaired two-tailed *t* test.

Given the apparent disparities in the rates of transformation between PCR products and gDNA, we were curious to directly compare transformation efficiencies between the two sources of DNA. Typically, if the source of DNA strongly impacts its transformability, it is because the organism can distinguish foreign DNA through its methylation patterns (Kobayashi, 2001) and/or through the presence of DNA uptake sequences (DUS) (Smith et al., 1999; Chen and Dubnau, 2003, 2004; Mell and Redfield, 2014) Thus, we extracted gDNA from a confirmed competence gene locus deletion mutant and compared its transformation efficiency with that of a PCR product harboring the same mutation. As shown in Figure 3A, gDNA transformation was extremely efficient with transformants comprising >0.1% of the total population. However, we observed a ~2-log lower transformation efficiency with a PCR product, despite the fact that the copy number of the PCR product was higher than the gDNA. This confirmed our previous observations, as both the pBSJL1 and PCR product transformations were far less efficient than gDNA transformation (Figures 1A, 2B,D). Since both the PCR product and gDNA targeted the same region of the chromosome and had identical sequences, it seemed unlikely that the disparate transformation efficiencies could be attributed to a DUS. For example, in *Haemophilus influenza, Neisseria meningitidis*, and *Neisseria gonorrhoeae*, DUS occur at an average frequency of 1/kb and even a single DUS is sufficient to facilitate uptake of both long and short DNA fragments (Mell and Redfield, 2014). If *V. parvula* exhibited a similar bias for DUS throughout its genome, we would expect our mutagenesis construct to contain at least one DUS, since it contained nearly 2 kb of sequence identical to the chromosome. To exclude the possibility that construct design was the source of the lower transformation efficiencies of PCR products, we created a series of additional mutations within the competence gene locus using PCR products harboring either small (~0.9 kb) or large (>2.5 kb) homologous fragments and creating either small (~0.4 kb) or large (~5.7 kb) deletions within the locus. Interestingly, in all cases, the PCR products yielded significantly fewer transformants than their corresponding gDNAs with most constructs yielding similar 10-fold lower transformation efficiencies relative to gDNA, indicating that construct design had little impact on the results (Figure 3A). The only exception was the first construct, which contained both small homologous fragments and created a large chromosomal deletion. It yielded an additional 10-fold reduction in transformation efficiency. Thus, construct design could be a partial contributor to the exceptionally poor transformability of this specific construct. We also repeated this experiment using our *recA* deletion construct and observed a similar preference for gDNA, which indicated that this phenomenon is unlikely to be locus-specific (data not shown). Since construct design did not appear to be the primary source of the *V. parvula* preference for genomic DNA, we were next interested to test the role of DNA methylation. If methylation were indeed a factor, we would predict significantly higher rates of transformation from plasmids purified from *V. parvula* vs. *E. coli*, due to differences in host methylation patterns. However, there appeared to be no obvious difference in the transformation efficiencies using plasmids purified from either *E. coli* or *V. parvula*. We did note substantially lower overall transformation efficiencies for pBSJL1 transformations compared to PCR products and gDNA (Figure 3B). Thus, for unknown reasons, *V. parvula* natural competence exhibits a clear hierarchy of preference for the source/type of DNA with plasmids being the least efficiently transformed and genomic DNA being the most efficient.

### Putative natural competence loci are widely encoded in the veillonellaceae family

**Figure 4:**
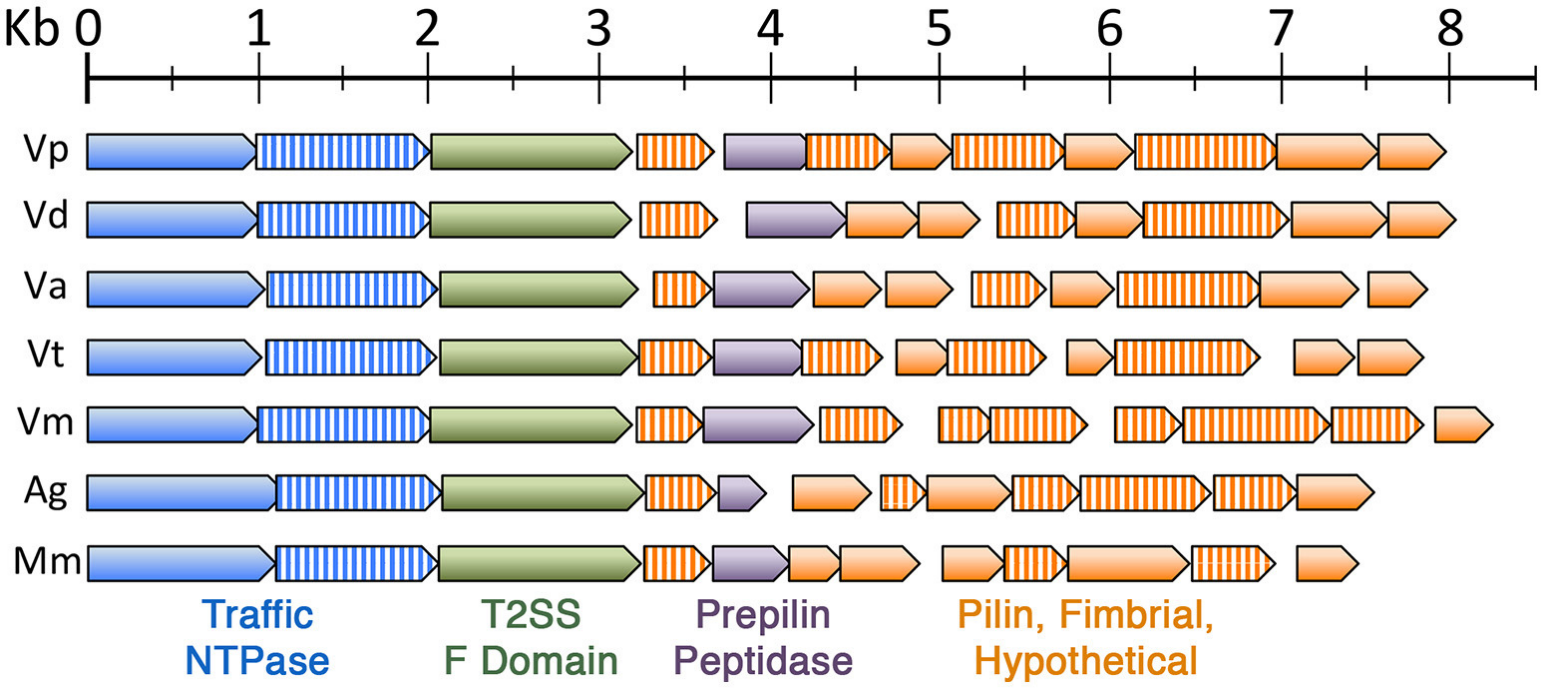
**The *V. parvula* competence locus is highly conserved in the Veillonellaceae family**. Chromosome maps of similar competence gene loci found among other members of the Veillonellaceae family. ORFs are all drawn to scale and are color coded as follows: blue (traffic NTPase), blue striped (PilT-type traffic NTPase), green (T2SS F domain protein), purple (prepilin peptidase), orange (pilin, fimbrial, or hypothetical), and orange striped (pilin, fimbrial, or hypothetical with a putative A24 prepilin peptidase cleavage/N-terminal methylation motif). The species and corresponding NCBI gene locus tags of putative competence gene loci are: *Veillonella parvula* (Vp, VPAR_RS05490—VPAR_RS05435), *Veillonella dispar* (Vd, VEIDISOL_RS04670—VEIDISOL_RS04725), *Veillonella atypica* (Va, HMPREF0870_00539—HMPREF0870_00528), *Veillonella tobetsuensis* (Vt, VEI_RS05560—VEI_RS05505), *Veillonella montpellierensis* (Vm, HMPREF0872_RS02280—HMPREF0872_RS02225), *Anaeroglobus germinatus* (Ag, HMPREF0080_RS06235—HMPREF0080_RS06175), and *Megasphaera micronuciformis* (Mm, HMPREF9429_RS07325—HMPREF9429_RS07270).

Given the relatively low efficiency of plasmid transformations in *V. parvula*, we decided to revisit our original natural competence screen of *V. parvula* isolates, as those transformations had utilized pBSJL1. Interestingly, when retesting these isolates using gDNA extracted from an SKV38 mutant, we were able to reliably transform more than half of our *V. parvula* isolates (Table 3). This suggested that natural competence is actually quite common among *V. parvula* strains, even when using low-passage clinical isolates. Consequently, we were curious whether similar competence gene loci might be found among other veillonellae. Indeed, this did prove to be the case, as the genes required for DNA uptake in *V. parvula* are widely conserved within the Veillonellaceae family. All of the putative competence loci found among these organisms exhibit synteny and encode two copies of traffic NTPases with the second copy bearing homology to *pilT* genes (Figure 4), which is highly suggestive of a similar role in DNA uptake.

**Table 3:** **Transformation of *V. parvula* strains with SKV38 mutant gDNA**.

***V. parvula* strain**	**Total CFU (10 μl)**
SKV1	<1
SKV9	35 ± 10
SKV10^*^	34 ± 13
SKV12	<1
SKV17B	23 ± 4
SKV21	32 ± 14
SKV24	<1
SKV31	5.56 × 10^3^ ± 2.64 × 10^3^
SKV38	8.11 × 10^3^ ± 3.47 × 10^3^
SKV43	<1
SKV44	<1
SKV65C^*^	<1
SKV66	9 ± 9

* *These strains are naturally tetracycline resistant and were therefore transformed with the same amount of a separate gDNA conferring spectinomycin resistance*.

*Data are presented as the means ± standard deviations from three independent experiments performed in triplicate*.

## Discussion

For many years, *Veillonella* was considered to be a genetically intractable genus, as attempts to transform different veillonellae were unsuccessful. This changed several years ago when it was demonstrated that electrotransformation was possible in *V. atypica* strain OK5 (Liu et al., 2012), which was soon followed by reports of targeted mutagenesis in this strain (Zhou et al., 2015a,b, 2016). In the current study, we provide the first evidence of natural competence for any species within the Veillonellaceae family and illustrate how this ability can be exploited for genetic studies. The key hindrance for observing natural competence among new species lies in the transformation procedure itself, as it is extremely difficult to predict the appropriate growth conditions required to trigger competence gene expression in species unknown to be naturally competent. For *V. parvula*, we fortuitously discovered that the combination of SK medium and agar plate growth serves as a reliable approach to activate its natural competence ability. While this approach functioned well for more than half of our *V. parvula* clinical isolates, there was still a wide range of transformability among the strains with nearly all strains clustering into one of three distinct groups: high competence (SKV31 and 38), low competence (SKV9, 10, 17B, and 21), and undetectable (SKV1, 12, 24, 43, 44, and 65C) (Table 3). Strain SKV66 was the lone exception, falling somewhere between the low competence and undetectable groups. It is currently unclear whether these differences in transformability are due to inherent strain-specific discrepancies in the levels of competence gene expression, differences in the environmental cues required to trigger competence development, or some combination of both factors. Regardless, medium composition was shown to be one of the major components triggering *V. parvula* natural competence development (Figure 1A). One of the principal differences in medium composition between SK and THL media is the lack of glucose in SK medium. Given the consistently higher competence observed in SK medium, it would appear that glucose could be a major inhibitor of *V. parvula* competence development. In *H. influenzae*, natural competence gene expression is critically dependent upon sugar uptake through the fructose phosphoenolpyruvate phosphotransferase system (PTS) (Macfadyen et al., 1996). Even though *Veillonella* species are classified as asaccharolytic (Delwiche et al., 1985), they still encode a variety of putative PTS transporters, including a putative fructose PTS transporter (VPAR_RS08070). Thus, it is conceivable that glucose (and perhaps other sugars) could be transported into the cell and inhibit competence gene expression. Growth on agar plates seems to be another fundamental aspect of *V. parvula* competence development. An agar plate transformation approach is also used to trigger natural competence in *Aggregatibacter actinomycetemcomitans* (Wang et al., 2002). Likewise, we have observed agar plate growth to be similarly stimulatory for natural competence in multiple species of *Streptococcus* (unpublished observations). We speculate that the high cell density and surface attached growth conditions on agar plates probably mimics the biofilm environment, which is known to be a major trigger for competence development in a diverse array of bacteria (Li et al., 2001; Meibom et al., 2005; Antonova and Hammer, 2011; Nishikawa and Tanaka, 2013).

One of the major advantages of natural competence for the transformation of genetic constructs is that the import of transforming DNA is coupled with the nucleolytic degradation of one of its strands (Chen and Dubnau, 2004). This protects newly imported DNA from restriction enzyme cleavage, as these enzymes specifically target dsDNA substrates (Bickle and Kruger, 1993; Kobayashi, 2001). In contrast, electroporation injects double stranded DNA into the cell, making it much more susceptible to restriction. Upon entering the cell single-stranded, naturally transformed foreign DNA can then be recombined with the host chromosome, resulting in temporarily hemi-methylated DNA at the site of recombination. Hemi-methylated dsDNA is also resistant to restriction enzyme cleavage (Kobayashi, 2001; Johnston et al., 2013). For this reason, it was quite surprising to observe such a stark preference for isogenic gDNA vs. PCR products with *V. parvula* natural transformation. It is currently unclear why this bias exists. Studies from naturally competent *Neisseria* and *Haemophilus* species indicate that DUS result in >100-fold increase in DNA uptake bias (Mell and Redfield, 2014), which is far greater than the bias we observed with most of our mutagenesis constructs (Figure 3A). In addition, we screened the genomes of *V. parvula* strains DSM 2008 and UTDB1-3 to identify overrepresented sequences that might serve as potential DUS. There are no sequences ≥10 bp represented ≥100 times in the genome, suggesting that any potential DUS would be likely be 9 bp or smaller (data not shown). There are >800 9 bp sequences represented ≥100 times in the genome (data not shown). Thus, more detailed studies would be required to determine whether any of these might serve as a DUS to improve DNA uptake. Initially, we suspected that the DNA uptake bias could be simply due to differences recombination efficiency, since gDNA transformations contain larger regions of homology for recombination. However, construct design only seemed to affect the efficiency of one of the constructs and all constructs yielded much fewer clones compared to gDNA including both of the constructs containing large homologous fragments (Figure 3A). This led us to suspect differences in DNA methylation status, since PCR products are unmethylated unlike gDNA. Similar observations have been made for both *Pseudomonas stutzeri* (Berndt et al., 2003) and *Helicobacter pylori* (Humbert et al., 2011) and the mechanism involved is still the subject of debate. It was recently proposed that this could be due to the lethal self-action of restriction enzymes targeting transiently unmethylated segments of non-homologous DNA found within the mutagenesis constructs (i.e., antibiotic cassettes, reporter genes, etc.) (Johnston et al., 2013). However, in our case, the evidence does not support a role for methylation (Figure 3B). For *V. parvula* plasmid transformations, the structure of the transforming DNA may be an additional contributing factor to its much lower efficiency, as most plasmid DNA is circular, whereas PCR products and gDNA are both linear.

Given the apparent poor transformability of plasmid DNA in *V. parvula* (Figure 3B), we suspect that our original lack of observable transformants in *V. atypica* and *V. dispar* is probably a consequence of using pBSJL1 for competence screening, since both species likely encode the same DNA uptake machinery as *V. parvula* (Figure 4). Therefore, as a general rule, it is probably advisable to screen for natural competence in bacteria using gDNA rather than plasmids, perhaps with gDNA derived from a spontaneous antibiotic resistant mutant. Furthermore, unlike most veillonellae, we were unable to identify similar competence loci in the genomes of *V. denticariosi* and *V. rogosae*, so these species may lack natural competence machinery altogether. Alternatively, their competence gene loci may simply be missing from the partial genome data available for these species. Furthermore, we have observed very similar type IV pilin/competence loci like those of the veillonellae to be widely distributed amongst numerous distantly related Gram positive and Gram negative organisms (data not shown). Thus, it is possible that natural competence is far more common among bacteria than is currently appreciated. Many of these organisms are also medically significant species that are largely recalcitrant for genetic manipulation. The results in the current study may serve as a useful template for the exploration of natural competence in these organisms.

## Author contributions

Data collection was performed by SK, CB, and JP. Data analysis and manuscript preparation were performed by FQ, JK, and JM.

### Conflict of interest statement

The authors declare that the research was conducted in the absence of any commercial or financial relationships that could be construed as a potential conflict of interest.
